# Nanocomposite 3D printed resins containing titanium dioxide (TiO_2_) nanoparticles: an *in vitro* analysis of color, hardness, and surface roughness properties

**DOI:** 10.3389/fdmed.2025.1581461

**Published:** 2025-05-21

**Authors:** Maram A. AlGhamdi, Shaimaa M. Fouda, Yousif A. Al-Dulaijan, Soban Q. Khan, Mai El Zayat, Raghad Al Munif, Zainab Albazroun, Fatma Hamza Amer, Ahmed Tharwat Al Ammary, Amr A. Mahrous, Mohammed M. Gad

**Affiliations:** ^1^ Department of Substitutive Dental Sciences, College of Dentistry, Imam Abdulrahman Bin Faisal University, Dammam, Saudi Arabia; ^2^ Department of Dental Education, College of Dentistry, Imam Abdulrahman Bin Faisal University, Dammam, Saudi Arabia; ^3^ College of Dentistry, Imam Abdulrahman Bin Faisal University, Dammam, Saudi Arabia; ^4^ Department of Family & Community Medicine, College of Medicine, Imam Abdulrahman Bin Faisal University, Dammam, Saudi Arabia; ^5^ Assistant Professor of Operative Dentistry Department, Faculty of Dental Medicine, Al-Azhar University, Assuit, Egypt

**Keywords:** 3D printing, complete denture, denture base resin, nanoparticles, optical properties

## Abstract

**Purpose:**

To investigate the effect of different titanium dioxide nanoparticle (TN) concentrations on the color stability and surface properties of additively fabricated (AF) denture base resins after thermal cycling.

**Methods:**

Two types of AF denture base resins, NextDent and ASIGA, were used to fabricate a total of 120 disc-shaped (10 × 2 mm) specimens (*n* = 10). The specimens of each resin were divided into 2 groups according to the concentration of titanium dioxide nanoparticles (1 wt.%, 2 wt.% TN) in addition to a control group of pure resin for each material. The specimens’ color change, hardness, and surface roughness (Ra) were tested after thermal cycling (5,000 cycles). Collected data was analyzed using ANOVA and *post hoc* Tukey's test (α = 0.05). The color change was referred to the National Bureau of Standards (NBS).

**Results:**

The addition of TN resulted in significant color changes in NextDent, with unacceptable changes according to the NBS (8.84 for 1 wt.% TN and 8.28 for 2 wt.% TN). In contrast, ASIGA showed significantly less color change than NextDent, and the changes remained within clinically acceptable limits. For hardness, in comparison to the pure group, TN addition didn't show any significant change in terms of TN concentrations and material type (*P* > 0.05), and the highest hardness value was recorded with NextDent/2 wt.%TN (16.6 ± 9.0 VHN). TN addition significantly increased Ra in NextDent, which was concentration-dependent (*p* = 0.001), while AISGA showed no change in Ra with TN addition (*p* = 0.693).

**Conclusion:**

Nanocomposite denture base resins containing TN increased the color change and surface roughness with no change in hardness. The effect of TN was material-dependent; therefore, resin material selection for nanocomposite preparation should be considered.

## 1 Introduction

The use of 3D printing technology in denture fabrication has increased due to multiple factors, including the ease of fabrication with fewer clinical appointments, increased patient satisfaction, and low cost (1). However, additively fabricated (AF) dentures lack the mechanical strength of milled dentures (2). Some studies reported comparable mechanical properties of additively fabricated denture base resin (DBR) to heat polymerized polymethyl methacrylate (PMMA) (3, 4), while others found inferior properties (5–8). The flexural strength, impact strength, and hardness of AF resin were found to be less than that of milled and conventional resin (2, 5, 7). The manufacturing technique of AF resin in layers could be the reason for its weak mechanical properties due to the weak bond between the layers (2, 5). On the contrary, milled resin is fabricated from prepolymerized PMMA blocks, and conventional heat-cured resin is fabricated in one piece using a mold technique, resulting in higher strength.

Different factors affect the strength of AF resin and are classified as pre-printing, printing, and post-printing factors (9). Modification of the resin fluid by nanoparticle addition prior to printing was previously investigated (10, 11). The obtained nanocomposites had a promising mechanical antibacterial performance and were recommended for 3D printed DBR fabrications (11–13).

TiO2 nanoparticles (TN) are biocompatible and recommended to be added to conventional (14–16) and 3D-printed resins (17–20), aiming to improve the properties of nano-modified resins. Previous studies tested the effect of TN with different concentrations ranging from 0.5 to 30 wt% and showed enhanced properties with the addition of 1–2 wt%, while above 5 wt% TN, the modified resin was weakened (15). Alrahlah et al. (14) reported improved mechanical and antimicrobial properties of PMMA after adding 1, 2, and 3% wt TN. AlGhamdi et al. (17) found that the addition of 1% and 2% TN increased the flexural strength of 3D-printed DBR with different post-curing times. Also, *C. albicans* adhesion to 3D-printed DBR was decreased after TN addition, which proves the antimicrobial activities of nanocomposites (18, 20). Altarazi et al. (19) also examined 3D-printed resins with varying TN concentrations (0.10, 0.25, 0.50, and 0.75 weight percent) after being aged in artificial saliva. They noted significant improvement of mechanical and physical properties, including flexural properties, impact strength, hardness, and degree of conversion (19).

The nanoparticles are required to improve the mechanical performance of the resin and its surface properties without altering the resin color (11, 21). The denture base material should match the color of the underlying gingiva and resist color change for acceptable esthetics (22). The surface roughness of denture base material is critical to avoid microbial colonization and staining. Hardness is another important property that makes the denture resist surface indentation (2). Some studies (10, 17, 19) reported alteration of surface properties by adding nanoparticles to AF DBR. Gad et al. reported increased hardness of 3D printed resin with the addition of nano-silica (NS) particles, though the surface roughness was not changed (10).

Studies that tested the effect of TN on the surface properties of AF DBR are limited. Moreover, as far as the authors’ knowledge, no previous studies have tested the effect of TN addition on the color of AF DBR. Therefore, this study aimed to test the effect of TN on the surface roughness, hardness, and color of two types of AF DBR. The first study hypothesis stated that adding TN to AF DBRs’ would not change the resin's color. The second hypothesis was that TN would not change the AF DBRs’ hardness or surface roughness.

## 2 Materials and methods

Based on sample size calculation, a total of 120 3D-printed specimens were fabricated. A power analysis was conducted to establish the study's sample size. The parameters for this calculation included a power of 80%, a confidence interval of 95%, and a significance level of 0.05. As a result, the determined sample size for each group was 10.

The materials used, their specifications, and printing procedures are detailed in Table 1. Two 3D-printed resins, NextDent and ASIGA, were used for specimen printing. For nanocomposite preparation, TN (30 m^2^/g surface area and 80–100 nm average sizes) was added to each fluid resin in two concentrations (1 wt.% and 2 wt.%), while one group remained unmodified as a control group resulted in 120 printed specimens ((60/resin, 30/Ra and color, 30/hardness). Before adding TN, the fluid resin container was placed on the shaker (NextDent LC-3DMixer, B. V., Soesterberg, the Netherlands) and shaken for 1 h according to the manufacturer's instructions. TN was weighed using an electronic balance, then added to each resin container, thoroughly mixed using a magnetic stirrer for 30 min, aiming to achieve a homogenous distribution of TN within the resin fluid.

**Table 1:** Materials composition, specifications, and specimen printing parameters.

Materials' specifications and printing process	NextDent	ASIGA
Brand name	Denture 3D + NextDent B.V., Soesterberg, The Netherlands	DentaBASE ASIGA, Erfurt, Germany
Compositions	Methacrylic oligomers, methacrylate monomer Bisacylphosphine oxide (BAPO) Phenyl bis (2,4,6-trimethylbenzoyl)-phosphine oxide Inorganic filler Pigments	Ethoxylated bisphenol A dimethacrylate 7,7,9 (or 7,9,9)-trimethyl-4,13-dioxo-3,14dioxa-5,12-diazahexadecane-1,16-diylbismethacrylate 2- hydroxyethylmethacrylate, Silicon dioxide Diphenyl (2,4,6-trimethylbenzoyl)-phosphine oxideTitanium dioxide
Nanocomposite	Titanium dioxide nanoparticles (Sigma–Aldrich, Co., St Louis, MO, USA) was added separately by 1% and 2% to each resin forming nanocomposite followed by shaking vigorously to confirm TN distribution within the resin fluid	
Specimens dimension	Disc-shape (10 × 2 mm)	
Printer	NextDent 5,100, NextDent B.V., Soesterberg, The Netherlands	ASIGA MAX™, ASIGA, Erfurt, Germany
Printing technology	DLP	
Wavelength	405 nm	385–405 nm
Post-curing machine	LC-D Print Box, NextDent B.V., Soesterberg, The Netherlands	ASIGA Flash, ASIGA, Erfurt, Germany
Post-curing time	30 min	20 min delivered [4,000 flashes (2 × 2,000 flashes each side)
Post-curing temperature	60°C	——

A disc-shaped (10 × 2 mm) specimen was designed using open-source AutoCAD software and was imported as a standard tessellation language (STL) file to two printers, NextDent and ASIGA. According to the manufacturer's recommendation, each container with nanocomposite mixture was shaken again for 1 h and then poured into the resin tank. This was followed by printing order with the following printing parameters: 50 µm printing layer thickness, and 90-degree printing orientation. After printing, the printed specimens were cleaned of unpolymerized resin remnants using 99.9% isopropyl alcohol. The additional polymerization in post-curing conditions was completed according to the manufacturer's recommendation (Table 1). After complete polymerization, all supporting structures were removed using a bur, followed by a conventional polishing technique. After support removal, the printed specimens were evaluated for voids, irregularities, or inappropriate dimensions, which were evaluated using a digital caliper. The approved specimens were polished conventionally, as described in the previous study with automated polishing machine (Metaserv 250 grinder-polisher; Buehler GmbH, Lake Bluff, IL, USA) using 1,200-grit sandpaper (MicroCut PSA; Buehler, IL, USA) for 5 min at 100 rpm in wet conditions (2) and detailed in Table 1. The specimens were maintained for 2 days in distilled water at 37°C before the thermal cycling ((Thermocycler, THE-1100/THE-1200, SD Mechatronik GMBH Miesbacher Str. 34 83,620 Feldkirchen- Westerham, Germany) for 5,000 cycles (where specimens immersed in water at 5°C and 55°C with a 30-second dwell time) to replicate six months of denture usage (23).

The CIE L*a*b* color space is commonly used to assess color changes in dental resins, with the 1976 CIE serving as an ISO/TR 28642:2016 standard reference (24). Color measurements were taken using a spectrophotometer (Color-Eye® 7000 A, X-Rite, Carlstadt, NJ, USA) operating in the visible spectrum (380–780 nm). The device was calibrated before each measurement using a white barium sulfate color round. The R reflection spectra of the samples were obtained with UVPROBE software version 2.21 (Shi-madzu Co., Kyoto, Japan). L*, a*, and b* were mathematically transformed using Color Analysis UV-2410PC, with all conversions performed under standard lighting conditions (CIE C). Then, the ΔE_ab_ formula was used to calculate the total color differences as described previously (25, 26). The National Bureau of Standards (NBS) was used as a reference for color change comparison and was calculated using the following equation: NBS = ΔE_ab_ × 0.92. A material is deemed aesthetically and clinically acceptable when NBS units fall within the range of 3.7 NBS units (25, 26). An NBS unit value exceeding 1 is detectable by the human eye. Differences greater than 3.7 NBS units are classified as a “mismatch” and are viewed as clinically unacceptable (25, 26).

The specimen's Vickers hardness (VH) was assessed with a hardness tester (HMV-2 Shimadzu Corp, Tokyo, Japan). A load of 50 g was applied for 15 s, and the average of three readings for each specimen was noted as the individual Vickers hardness (VH) (2).

A noncontact Profilometer (Contour GT-K 3D Optical Microscope, Bruker, Billerica, MA, USA) with 0.38 µm lateral resolution was used to measure each specimen's average surface roughness (Ra) in three randomly selected areas (27, 28).

The Shapiro–Wilk test indicated that the data followed a normal distribution. Therefore, parametric tests were utilized for inferential analysis. Two-way ANOVA was employed to examine the interaction effects of material type and NP concentration levels on the tested properties. To compare the mean differences among categorical variables with more than two categories, one-way ANOVA was conducted, followed by Tukey's *post hoc* test. A *p*-value of less than 0.05 was deemed statistically significant.

## 3 Results

Two-way ANOVA results for all tested properties are summarized in Table 2. The intercept showed significant differences for all tested properties (*p* < 0.001), while the combined effect of material type and TN concentration showed non-significant differences for all tested properties (*P* > 0.05).

**Table 2:** Two-way ANOVA results for all tested properties in terms of nanoparticles, concentrations, and material combinations.

Tested properties	Source	Type III sum of Squares	Df	Mean square	F-value	*P*-value
ΔE_ab_	Intercept	1,609.225	1	1,609.225	1,979.609	<0.001*
	Material *TN %	1.736	1	1.736	2.135	0.147
	Error	87.793	108	.813		
	Total	2,545.717	120			
Hardness (VHN)	Intercept	33,468.804	1	33,468.804	1,567.291	<0.001*
	material * TN %	283.423	1	283.423	13.272	0.346
	Error	2,306.292	108	21.355		
	Total	37,084.646	120			
Surface roughness (Ra, µm)	Intercept	220.841	1	220.841	2,517.650	<0.001*
	material * TN %	.022	1	.022	.245	0.621
	Error	9.473	108	.088		
	Total	246.783	120			

* Statistical significance at 0.05 level of significance.

**Figure 1:**
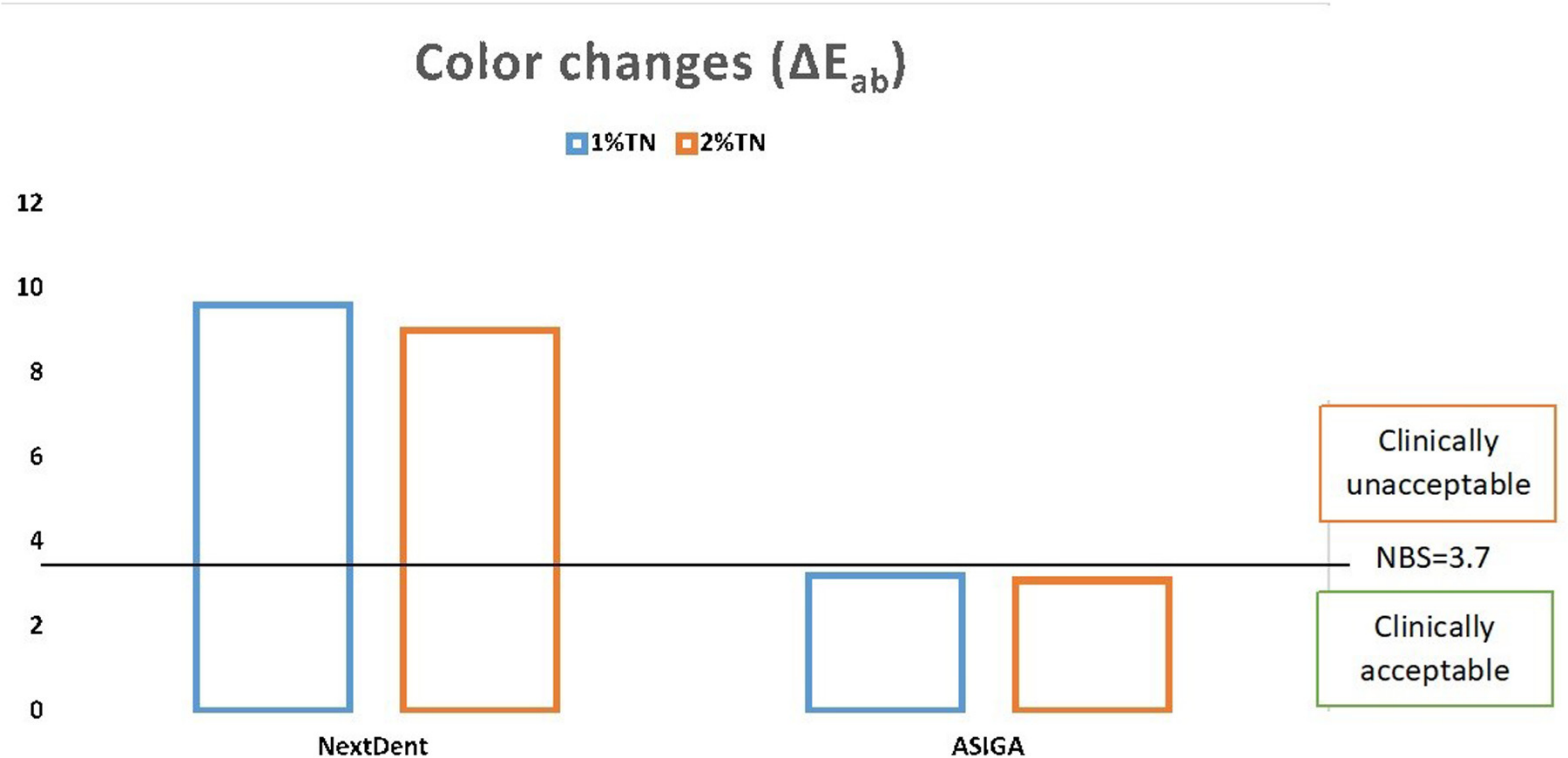
Color change values and NBS reference.

The mean, SD, and significances of the color change between groups in relation to concentration are presented in Table 3. The color change was higher with NextDent (*P* < 0.001) than with ASIGA. The difference between the two tested concentrations on color change was not statistically significant. The color change values of NextDent groups were above 3.7 NBS, TN with NextDent at both concentrations 1 wt.% (8.84 NBS) and 2 wt.% (8.28 NBS), while for ASIGA, the NBS values were below 3.7 (Figure 1).

**Table 3:** Mean and SD of color change (ΔE_ab_) of TN groups.

Materials	TN % (mean ± SD)				*P* value
	1%	NBS	2%	NBS	
NextDent	9.61 (1.26)	8.84	9.0 (1.01)	8.28	0.255
ASIGA	3.2 (0.8)	2.94	3.1 (0.8)	2.85	0.823
*P* value	*p* < 0.001*		*p* < 0.001*		

For NBS, values above 3.7 NBS units are rated a “mismatch” and considered clinically unacceptable.

* *p* < 0.05 significant difference.

Table 4 summarizes the mean, SD, and significance between groups concerned with the TN concentration's effect on the hardness of the tested resins. For NextDent, TN addition showed no significant difference in hardness (*P* = 0.735). Meanwhile, for ASIGA, 2 wt.%TN showed lower hardness than pure and 1 wt.%TN (*p* = 0.046). Compared to the material, there are no significant differences in hardness between NextDent and ASIGA with TN addition.

**Table 4:** Mean and SD of hardness and surface roughness of pure and TN-modified tested groups.

Tested properties	Materials	TN wt.% (mean ± SD)			*P* value
		Pure	1%	2%	
Hardness (VHN)	NextDent	14.58 (3.7)	16.5 (3.7)	16.6 (9.0)	0.735
	ASIGA	14.1 (4.3)^a^	14.1 (4.3)^a^	11.94 (3.4)	0.046*
	*P* value	0.275			
Surface roughness (µm)	NextDent	1.5 (0.24)	1.87 (0.4)	2.29 (0.3)	0.001*
	ASIGA	1.57 (0.27)	1.33 (0.4)	1.4 (0.3)	0.693
	*P* value	0.001*			

^a^ The same small letter per raw indicates non-significant differences between groups.

* *p* < 0.05 significant difference.

Table 4 includes the mean, SD, and significance of the surface roughness between groups in relation to TN concentration. The Ra of NextDent specimens was increased by TN addition with both concentrations (*P* < 0.001). For ASIGA, the effect of concentration on Ra was not statistically significant. NextDent showed higher Ra than ASIGA in all tested groups.

## 4 Discussion

This study aimed to assess how adding TN affects the color change, hardness, and surface roughness of two commercially available 3D-printed resins following artificial aging through thermal cycling (5,000 cycles). The first study hypothesis is rejected, as the addition of TN to NextDent increased the color change at both concentrations above the clinically acceptable threshold. In contrast, the color change for ASIGA was considered acceptable. The hardness of both materials was not affected by TN addition except ASIGA at 2%TN, while the surface roughness of NextDent was increased at both TN concentrations. Accordingly, the second study hypothesis is partially accepted.

The denture base is subjected to hot and cold environments in the oral cavity and is continually exposed to moisture (23). This environment allows water sorption, impacting the DBR's strength (19). The amount of water sorption increased with temperature, so during the hot cycle, the amount increased, and the absorbed water acted as a plasticizer, which affected the strength of printed resin (29). In addition, the absorbed water may contain stains that directly affect DBRs’ color. Moreover, the accumulated water macule affects the translucency of DBR due to its ability to produce light refraction. Therefore, thermal cycling for all specimens was done for 5,000 cycles, simulating 6 months of clinical use (23).

TN is a biocompatible nanoparticle that is recommended to be added to PMMA DBRs, and it has shown some promising effects (15). Similarly, TN added to 3D-printed DBRs showed antifungal effect and improved the mechanical performance of 3D-printed nanocomposites (17–20). Altarazi et al. (19, 20) added low TN concentrations (0.10, 0.25, 0.50, and 0.75 wt.%) to 3D-printed resins. They found that incorporating a low percentage of TN increased the mechanical strength. At the same time, clusters are formed with increased TN concentration, decreasing the mechanical performance of 3D printed resins (19). Altarazi et al. (20) proved that 0.5 wt.%TN showed a profound reduction in *C. albicans* adhesion and recommended this nanocomposite for denture base fabrications with antifungal activity. Totu et al. (18) added high concentrations (0.2, 0.4, 0.6, 1, 2.5 wt.%) of TN and found that specimens containing 0.4, 1, and 2.5wt.%TN inhibited the growth of Candida on nanocomposite specimens surface. AlGhamdi et al. (17) added 1% and 2% TN and found increased flexural strength as TN increased. Due to the variation in concentrations used in previous studies, the high concentrations have more impact on strength, and high concentrations (1 wt.% and 2 wt.%) were selected in the present study. Although a few previous studies evaluated the effect of TN on the properties of 3D-printed DBRs, there is still a lack of studies investigating the effect of TN on color properties and related surface properties. Therefore, two concentrations of TN were added to two different 3D-printed resins.

The color change in this study was evaluated using CIE L*a*b*, which has been commonly used to evaluate the color change of dental resins (24–26). NBS values of ASIGA are within the clinically acceptable value, while NextDent showed an unacceptable value regardless of the TN concentration. This finding confirmed that the material type with TN addition affected the color change. The difference in material compositions might be the cause of this variation. Due to the lack of previous studies investigating the color of 3D-printed nanocomposites containing TN, the comparison with previous studies was difficult. However, TN-modified DBRs were used for comparisons. It was expected that the addition of TN would significantly affect the color of DBRs due to the opacity of TN, and its white color resulted in a whitish color of the nanocomposite (16). It was reported that the color change by TN addition is due to the difference between the resin and TN refractive indices, leading to increased opacity of the nanocomposite (30). The difference between TN and resin refractive indices is high; the alteration in color was related to material composition, regardless of whether TN was added. This may be attributed to the low concentrations used in the present study compared to previous studies, which used 2.5 wt.% and 3 wt.% (16, 31) up to 7 wt.% TN (32).

Regarding material compositions, NextDent is an ester-based polymer with more inorganic fillers and pigments added to the resin matrix, and all of these components are the reason for the color change of NextDent (unacceptable color). On the other side, ASIGA showed fewer color changes, and all values are within the clinically acceptable range. This finding recommends using ASIGA in the case of TN used for 3D-printed nanocomposites. ASIGA contains titanium dioxide particles, according to the manufacturer's claim, and it was expected that the effect of TN addition would increase the amount of TN per specimen. Finally, the effect was supposed to be more in ASIGA with TN addition. The presence of fillers and pigments affected the refraction and absorption of light during specimen testing (33). This also confirms that material composition has a role in color, regardless of the TN addition and concentrations. Khattar et al. (34) found similar results, examining the translucency of 3D-printed DBRs with and without zirconia (ZrO_2_) nanoparticles. They reported that the ASIGA group exhibited greater translucency compared to the NextDent group.

Hardness represents the material's resistance to abrasion. Material with high resistance is recommended for denture longevity; otherwise, more abrasion would increase roughness and stainability, which would deteriorate the denture esthetics (2). In the current study, the hardness was not affected by TN at both concentrations, and with both materials, except for 2 wt.% ASIGA; a significant decrease was observed. The unchanged hardness with TN addition to NextDent is in agreement with Al Ghamdi et al. (17) findings that tested the same TN concentrations (1 wt.% and 2 wt.%) as in this study but with different parameters. On the contrary, Altarazi et al. (19) reported that TN addition increases the hardness of 3D-printed resin. However, they tested a lower concentration of TN than was used in our study, which might be the cause of this variation. It was reported that adding more fillers to 3D-printed resin increases the viscosity, which could affect the printing and polymerization process (35, 36).

Additionally, the filler concentrations may affect the degree of monomer conversion, leading to a higher percentage of residual monomer, which inversely affects the strength of the printed object (19, 35, 37). In thermal cycling procedures, the residual monomer leaches out, allowing more water sorption, and the amount of absorbed water acts as a plasticizer that weakens the surface of materials. In the case of ASIGA 2 wt.%TN, the decrease in hardness may be due to the increased amount of TN nanoparticles per specimen, as the original material contains TiO_2_ within its composition. The increased TN amount form clusters and the agglomeration of these clusters on the specimens’ surface may be another explanation for the hardness decrease (17, 37). Moreover, these clusters inside the resin matrix act as stress concentration areas, deteriorating the material's internal structure (11).

The surface roughness of DBRs is one property that affects other properties, such as color stability and microbial adhesion (38). High roughness resulted in more microbial adhesion and biofilm formation and more adherence to stains and discoloration (38). Therefore, investigating the surface roughness of the introduced nanocomposite was the focus of the current work. According to its findings, the Ra of ASIGA didn't change with TN addition, while the TN addition increased the Ra of NextDent, and the increase was concentration-dependent (as the % increased, the Ra increased). The increase in Ra may be attributed to the presence of some TN clusters on the specimens’ surface (15, 16, 39). In disagreement, a previous study (16) reported that the Ra of PMMA was not affected by the addition of different concentrations (1 wt.% and 2.5 wt.%) of TN. Hence, they used a higher concentration than the present study. The difference in findings may be related to the difference in methodology and the double-layer technique used for PMMA denture base fabrication, which differed from the printed resin used in the present study.

The minimum clinically acceptable Ra threshold is 0.2 µm. Levels above this threshold lead to increased microbial adhesion (40). When comparing the Ra values of the present study, all values of unmodified and modified 3D-printed DBRs are above the minimum Ra clinically acceptable value. Gad et al. (5) reported higher surface roughness of 3D-printed resins after thermal cycling compared to PMMA and CAD-CAM denture base materials. They attributed this increase to the printing nature, layer-by-layer, and the stepwise effect between the two successive printed layers (5). Therefore, when TN is added to 3D-printed DBRs, other parameters should be considered to get a 3D-printed nanocomposite with appropriate properties (11). In addition, obtaining a smoother surface of the 3D-printed resin could be achieved by the specimens’ surface treatment, using different polishing techniques, and/or surface coating with Nano ceramic resin coatings.

From a clinical point of view, the addition of TN altered the color and hardness of NextDent, and ASIGA showed no significant change in the aforementioned properties. This may be due to the difference in the composition of the materials. The material type showed variations between the tested properties regardless of TN addition; therefore, material selection for reinforcement should be considered when TN is selected as an additive. However, further research is necessary to optimize the TN content for optimal color and surface properties while maintaining mechanical integrity.

Hence, thermal cycling is considered a strength point of this study. Limitations of the study are due to the absence of other intraoral factors, such as occlusal forces and oral flora, in the *in vitro* setting of this study. In addition to other limitations related to specimen configuration, disc-shaped instead of denture configurations, and a lack of specimen imaging to confirm TN dispersion. Therefore, future studies in conditions simulating the oral environments with real denture configurations are recommended, using scanning electron microscopy to detect TN distribution.

## 5 Conclusions

Within the study's limitations, the following can be concluded:

The effect of TN addition varied between the tested materials; it caused a significant color change in NextDent that exceeded acceptable values, increased its surface roughness, and did not change the hardness. At the same time, the color change of ASIGA was within the acceptable clinical threshold. The surface roughness remained unchanged, and the hardness decreased with 2% TN addition. The tested properties showed variations between NextDent and ASIGA, highlighting the influence of material type and/or printing technology on the properties of the nanocomposite.

## Data Availability

The original contributions presented in the study are included in the article/Supplementary Material, further inquiries can be directed to the corresponding author.
